# Flavodoxins as Novel Therapeutic Targets against *Helicobacter pylori* and Other Gastric Pathogens

**DOI:** 10.3390/ijms21051881

**Published:** 2020-03-10

**Authors:** Sandra Salillas, Javier Sancho

**Affiliations:** 1 Biocomputation and Complex Systems Physics Institute (BIFI)-Joint Units: BIFI-IQFR (CSIC) and GBsC-CSIC, University of Zaragoza, 50018 Zaragoza, Spain; sandrasalillasberges@gmail.com; 2 Departamento de Bioquímica y Biología Molecular y Celular, Facultad de Ciencias, University of Zaragoza, 37009 Zaragoza, Spain; 3 Aragon Health Research Institute (IIS Aragón), 50009 Zaragoza, Spain

**Keywords:** flavodoxin, drug discovery, therapeutic target, *Helicobacter pylori* (*Hp*), antimicrobial resistance, gastric pathogens, gastric microbiota

## Abstract

Flavodoxins are small soluble electron transfer proteins widely present in bacteria and absent in vertebrates. Flavodoxins participate in different metabolic pathways and, in some bacteria, they have been shown to be essential proteins representing promising therapeutic targets to fight bacterial infections. Using purified flavodoxin and chemical libraries, leads can be identified that block flavodoxin function and act as bactericidal molecules, as it has been demonstrated for *Helicobacter pylori* (*Hp*), the most prevalent human gastric pathogen. Increasing antimicrobial resistance by this bacterium has led current therapies to lose effectiveness, so alternative treatments are urgently required. Here, we summarize, with a focus on flavodoxin, opportunities for pharmacological intervention offered by the potential protein targets described for this bacterium and provide information on other gastrointestinal pathogens and also on bacteria from the gut microbiota that contain flavodoxin. The process of discovery and development of novel antimicrobials specific for *Hp* flavodoxin that is being carried out in our group is explained, as it can be extrapolated to the discovery of inhibitors specific for other gastric pathogens. The high specificity for *Hp* of the antimicrobials developed may be of help to reduce damage to the gut microbiota and to slow down the development of resistant *Hp* mutants.

## 1. Introduction

*Helicobacter pylori* (*Hp*) is a Gram-negative, spiral-shaped bacterium that colonizes the gastric mucosa of over 4 billion people worldwide [1,2,3]. The prevalence of this infection increases with age and varies depending on the world region, being higher in developing countries (up to 88%) than in developed ones [2,3,4]. It is suggested that the HLA-DQA1 gene influences the human susceptibility to *Hp* infection, the development of related diseases, and the host’s response against this bacterium [5]. The ways in which *Hp* is acquired are proposed to include intake of contaminated water and direct human–human contact [3]. Diet, hygiene, and lifestyle play an important role in *Hp* transmission [2], and unless antimicrobial therapy is administered, humans can remain infected for life [3]. Although most infected people are asymptomatic [3], *Hp* colonization of the gastric epithelial cells can cause an inflammatory response in the mucosa. The initial gastritis can progress to chronic non-atrophic, active or atrophic gastritis and lead to duodenal and gastric ulcers or even to intestinal metaplasia and dysplasia, occasionally causing gastric mucosa-associated lymphoid tissue (MALT) lymphoma or gastric adenocarcinoma [3,6]. In fact, *Hp* is the only bacterium classified as a Class I carcinogen by the International Agency for Research on Cancer [3,6,7] and, as shown by epidemiological studies, it seems to be the most common infectious agent related to cancers, 6.2% of all cancer cases worldwide being attributable to *Hp* [6,8]. The risk of developing *Hp*-related cancer has been suggested to depend on the *Hp* strain, the host traits, and the interactions between bacterium and host [9]. Besides, *Hp* has been reported to be involved in extragastric pathologies such as neurological, dermatological, hematologic, ocular, cardiovascular, metabolic, allergic, liver, and biliary diseases [10,11]. The eradication of *Hp* has been recommended in order to decrease gastric mucosa inflammation and to prevent its progression to preneoplasic lesions and the development of gastric cancer and/or other extragastric diseases [12,13].

Conventional treatment of *Hp* infection has relied on two or three broad-spectrum antimicrobials plus a proton-pump inhibitor (PPI) such as omeprazole, esomeprazole or rabeprazole. Although standard triple therapy, which is based on clarithromycin, amoxicillin, or metronidazole and a PPI, has been prescribed for decades, nowadays it does not accomplish acceptable eradication rates because of *Hp* resistance, especially to metronidazole and clarithromycin. In areas of high (>15%) resistance to the latter antibiotic, bismuth or non-bismuth quadruple regimens must be followed. They consist of a PPI plus three antimicrobials: metronidazole, tetracycline, and bismuth in the first therapy and metronidazole, amoxicillin, and clarithromycin in the second one [14,15]. These last regimens seem the most effective ones to overcome antibiotic resistance, the main proposed reason of treatment failure together with low patient compliance to therapy, high gastric bacterial load, cytochrome P450 polymorphism (CYP2C19), and high gastric acidity [16]. Antibiotic resistance to *Hp* has been suggested to arise from point mutations, drug inactivation, the activation of drug efflux pumps, altered membrane permeability, biofilm formation or the presence of bacterial dormant forms [17]. The high genetic diversity of *Hp* allows the bacterium to evade the immune response and to adapt to environment challenges such as antimicrobials [18,19]. The annual *Hp* reinfection rate is up to 8.7% and depends on world region, age, education level, proportion of household members infected, and socioeconomic status of the patients [12]. While the reported prevalences of amoxicillin (0–21.4%) and tetracycline (0–32.4%) resistance are moderate, those of metronidazole (2.1–99.5%), clarithromycin (7.9–52.6%), and levofloxacin (0–55.6%) are quite high [12]. In fact, clarithromycin-resistant *Hp* strains were included by the World Health Organization in the high-priority group of pathogens that urgently require novel treatments [20]. Additional therapeutic regimens have been proposed that include the use of vonoprazan, furazolidone, rifabutin, fluoroquinolones, and probiotics-containing treatments [12,13,15,21,22]. Recent works suggest that therapies against *Hp* should be adapted to local antibiotic resistances, and the Maastrich V/Florence consensus report recommended, after failure of second-line treatment, culture with susceptibility testing or molecular determination of genotype resistance [13,15,21,22,23]. While prophylactic or therapeutic vaccines for *Hp* have been investigated, no vaccine has been developed yet, probably because of high *Hp* genetic variability together with the fact that the infection downregulates the host’s immune response which highlights the importance of selecting *Hp* antigens and adjuvants capable of triggering a strong host immune reaction [24,25]. Several novel therapeutic strategies for the treatment of *Hp* infection have been suggested including phototherapy and the use of antimicrobial peptides, gastric mucins, polysaccharides or bioactive compounds [24]. Related to the use of novel bioactive compounds, key *Hp* gene products have been proposed for directed therapies [26]. One of them is flavodoxin [27,28], a small electron transfer protein involved in an essential *Hp* metabolic pathway. Flavodoxin is also expressed in other gastrointestinal pathogens and also in human gut commensal bacteria. As it is essential for some commensal bacteria [29], it is important to develop flavodoxin-based therapies that are not harmful to these microorganisms in order to avoid side effects on the gastrointestinal microbiota. On the other hand, as flavodoxin is also essential for several gastrointestinal pathogens, this protein constitutes a useful target for developing specific treatments against them.

In this review, we compile and discuss *Hp* proteins that may act as potential targets with a special focus on the properties of flavodoxin that make it a promising therapeutic target for treating this infection. We then summarize ongoing efforts to develop *Hp*-specific flavodoxin inhibitors, and, finally, we discuss the possibility of extrapolating them to target the flavodoxins of other gastric pathogens for the treatment of the corresponding infections.

## 2. Targets for *Hp* Infection

**Figure 1:**
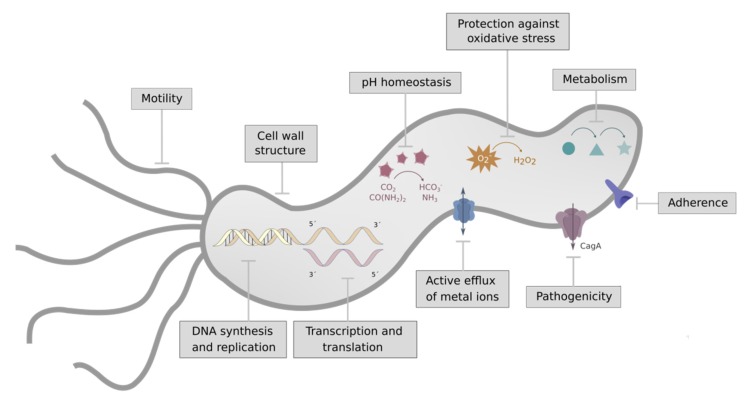
Pathways providing therapeutic targets to fight *Helicobacter pylori* infection.

Because resistance to currently used antibiotics in *Hp* infection is widespread, new antimicrobials targeting bacterial functions different from the classically targeted ones (e.g., cell wall integrity, nucleic acid synthesis and replication, or transcription and translation) are required. The new targets must be essential for bacterial survival or important factors for colonization or virulence, and they should be absent in humans so that toxicity risk is minimized. Complying with those requisites, several *Hp* pathways (Figure 1) have been proposed for the development of new drugs. Some of them are detailed in Table 1, and the more relevant ones are described below.

### 2.1. Metabolism

The shikimic acid pathway uses erythrose-4-phosphate and phosphoenol pyruvate to produce chorismic acid, the precursor of aromatic amino acids, folate cofactors, ubiquinone, and vitamins E and K. This biosynthetic route involves four *Hp* essential enzymes that are absent in mammals: 3-dehydroquinate dehydratase, shikimate dehydrogenase, shikimate kinase, and chorismate synthase [30,31,32,34]. The biosynthesis of coenzyme A (CoA), an essential bacterial cofactor, is achieved with participation of phosphopantetheine adenylyltransferase (PPAT), the inactivation of which prevents bacterial viability [35,36]. Fumarate reductase is a key enzyme for aerobic and anaerobic respiration which contains three subunits: FrdA, FrdB, and FrdC. Some fumarate reductase inhibitors used to treat helmintic infection have also shown inhibitory and bactericidal properties against *Hp* [33,38]. Several other *Hp* enzymes have been associated with bacterial respiration, rendering them critical for bacterial survival. Some of them: cytochrome c-type biogenesis protein CcdA and cytochrome c oxidase subunits CcoN, CcoO, CcoP, and CcoQ have been described as potential drug targets for *Hp* infection [37]. Enzymes and electron carrier proteins that take part in pyruvate decarboxylation (such as flavodoxin (Fld), pyruvate:flavodoxin oxidoreductase (POR), and flavodoxin:quinone reductase B (FqrB)) have also been identified as essential proteins for *Hp* survival [26,28,41,42,43,44,45,46].

### 2.2. Cell Wall Structure

Peptidoglycan, synthesized by Mur enzymes in a multistep pathway, is an essential component of the bacterial cell wall. MurA, acting on the first step, is targeted by Fosfomycin [50]. For glutamate racemase MurI, transforming L-Glu into D-Glu, several inhibitors have been described [49,50]. The widely used β-lactam antibiotics inhibit peptidoglycan cross-linking which is carried out in the last steps of the pathway [17,49,50]. On the other hand, the succinylase pathway is the only route in *Hp* for the synthesis of lysine, a required element of the bacterial peptidoglycan cell wall. N-succinyl-L,L-diaminopimelic acid desuccinylase (SDAP-deacylase; DapE), an enzyme of this route, has been identified as essential for *Hp* survival [47,48].

### 2.3. pH Homeostasis

*Helicobacter pylori* is able to survive the acidic pH in the stomach thanks to, at least, two enzymes playing a fundamental role in acid acclimation. Urease and carbonic anhydrase maintain neutral pH in the bacterial cytoplasm and periplasm by converting urea and carbon dioxide into ammonia and bicarbonate [54,55,67]. Urease, which is absent in humans, is a critical enzyme for *Hp* colonization of the host stomach. It is composed of α (UreA) and β (UreB) subunits and its activity requires Ni (II) ions [53,54,56]. The putative nickel-responsive regulator (NikR) regulates urease expression and nickel uptake [54]. This metal goes into the cytoplasm, where urease is localized, through the nickel–cobalt transporter NixA [54,59], and its incorporation to the active sites of urease taking place during enzyme maturation depending on UreD, UreE, UreF, UreG, and UreH accessory proteins [54,59,61,62,63] and on the HypA and HypB hydrogenase/urease maturation factors [61,63]. Conversely, HspA and Hpn proteins are related to nickel homeostasis, storage, and protection from higher concentrations of metal ions [59,61,64]. The access of urea to urease is restricted by an H^+^-gated pore (UreI) which regulates the urea entry into the cytoplasmic space [66]. The activity of these proteins is needed for urease function and, thus, for *Hp* colonization, and some of them (specifically, urease and HspA) can be inhibited by bismuth, an antimicrobial currently used in therapy [59]. On the other hand, two different types of carbonic anhydrase, α- and β-, have been identified in *Hp* periplasm and cytoplasm, respectively. Both metalloenzymes have been described to be essential for acid acclimation, biosynthetic reactions, bacterial survival, and colonization of the stomach and duodenum [67,68,70,71,72]. They are targeted by sulphonamide antimicrobial agents and phenol-derivatives [68,69,71].

### 2.4. Virulence (Adherence, Motility, and Pathogenicity)

*Helicobacter pylori* has developed structures and mechanisms contributing to bacterial virulence. Among them are adhesins, pili, flagella, and extracellular polymeric matrix materials such as DNA, polysaccharides, proteins, and lipids [96]. Biofilms are seen as virulence factors, and several mucoactive and antibiofilm substances, such as N-acetylcysteine and erdosteine, have been proposed as new adjuvant agents for therapy [96,97,98]. Motility, a crucial virulence factor needed for persistent *Hp* infection, is provided by flagella which allow bacteria to travel through the mucus layer from the gastric lumen to the epithelial surface, its site of infection. Flagellar filaments consist of the major (FlaA) and the minor (FlaB) flagellin proteins [53,75,76,78]. Additional related genes, *fliD* and *flgK*, are required for flagellar filaments assembly and flagella formation [76,77,78]. For flagellar assembly and motility, flagellin needs to be O-glycosylated with pseudaminic acid (Pse). Thus, the Pse biosynthesis pathway and, in particular, the aminotransferase enzyme PseC have potential as targets [73,74]. Severe complications of bacterial infection have been related to *Hp* adherence to host cells. Secretion of virulence factors, such as those encoded in the cytotoxin-associated gene pathogenicity island *cag*PAI (CagA: the cytotoxin-associated antigen, and T4SS: the *cag*-type IV secretion system), the vacuolating cytotoxin VacA, or the blood group antigen-binding adhesin BabA, have been related to increased epithelial damage and predisposition to gastric carcinogenesis [82]. In particular, BabA enables bacterial contact with the stomach mucosa. Then, VacA delivery and T4SS signaling are induced: VacA leads to membrane pore formation, cellular vacuolation, apoptosis, and inhibition of immune cells [85,86], while the T4SS pathway translocates CagA into host epithelial cells, where it modulates aspects of the host metabolism and provokes inflammation, metaplasia, and neoplastic transformations [80,81,82,83,86]. The adhesin BabA has been associated to disease-related strains and *cag*PAI and VacA have been linked to increased gastric cancer risk [81,86]. On the other hand, the T4SS route is favored by interaction between the adhesin HopQ and the human carcinoembryonic antigen-related cell adhesion molecules (CEACAMs) [84]. In addition to those indicated above, other virulence factors have been identified in *Hp* which include adhesins, such as HopZ, OipA, SabA, and AlpA/B [76,80,85], and the HtrA serine protease, an essential periplasmic protein with chaperone and proteolytic activities involved in quality control and stress responses [85]. Specifically, HtrA is involved in the cleavage of the tumor suppressor E-cadherin and so in the disruption of intercellular adhesion and access of bacteria to intracellular spaces [85,87,99,100].

### 2.5. Active Efflux of Metal Ions

The levels of cobalt, zinc, cadmium, and iron need to be regulated in *Hp* as both too low and too high concentrations can be detrimental for bacterial life. Thus, enzymes that control metal levels (such as cation efflux system protein CusA, cobalt/zinc/cadmium efflux system membrane fusion protein, cobalt/zinc/cadmium resistance proteins CzcA, CzcB, and CzcC, CznABC metal efflux pump, ferrix siderophore transport system TonB periplasmic binding protein, ferric siderophore transport system ExbB biopolymer transport protein, and Haemin uptake system ATP binding protein) are crucial for *Hp* virulence and adaptation to gastric environment and, therefore, for bacterial survival [37,89].

### 2.6. Protection against Oxidative Stress

Toxic reactive species can cause oxidative stress to *Hp*, leading to cell death. For this reason, bacterial gene products involved in protection against reactive oxygen species, superoxide, and free radicals have been proposed as therapeutic targets. They include glutathionyl spermidine synthetase, iron-binding ferritin-like antioxidant protein, DNA-binding protein Dps, and superoxide dismutase [37]. Homeostatic stress regulator (HsrA) is an orphan response regulator unique among epsilonproteobacteria. It syncs metabolic functions and virulence with availability of nutrients and cell division. This protein regulates its own expression and that of a large number of genes involved in transcription, translation, energy, and nitrogen metabolism as well as redox homeostasis and oxidative stress defense. Due to the fact of its essentiality, its absence in humans and the availability of an X-ray structure, it has been proposed as a promising therapeutic target against *Hp* [92,93,94,95].

As can be seen, several *Hp* gene products have been described that may constitute appropriate targets for the development of novel therapies against this bacterial infection. Among them, flavodoxin stands out as a promising candidate due to the fact of its essentiality for *Hp*, its absence in humans, and because of structural features that will be discussed below. Stimulated by these facts, our laboratory has undergone several studies aimed at understanding the biophysical properties of *Hp* flavodoxin and to identify and perfect molecules that could bind to it and interfere with its vital function. The following sections include a detailed description of this flavodoxin, focusing on the properties that make it a fine drug target.

## 3. An Overview of Flavodoxins and of the Flavodoxin from *Hp*

**Figure 2:**
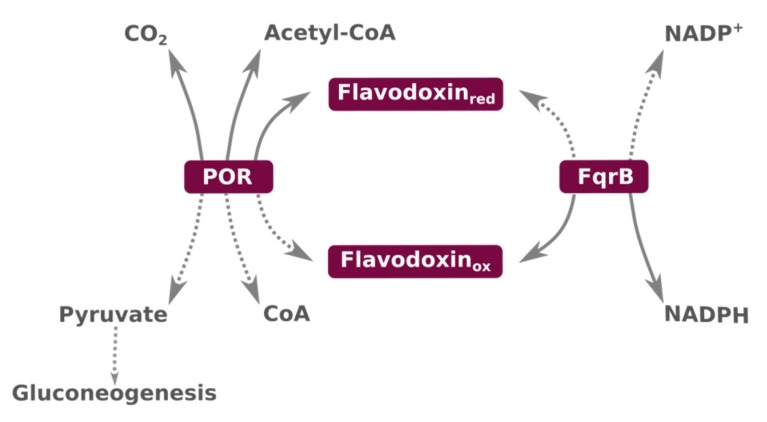
The POR:FldA:FqrB pathway in *H. pylori*. Flavodoxin (Fld) shuttles electrons between pyruvate oxidoreductase complex (POR) and flavodoxin:quinone reductase (FqrB) in a reversible pathway which plays a central role in the bacterial metabolism, as it represents an essential route for CO_2_ fixation and pyruvate metabolism. The pyruvate decarboxylation pathway is represented by solid lines, whereas the pyruvate synthesis pathway (contributing to gluconeogenesis) is indicated by dotted lines. Adapted from Reference [46].

Flavodoxins are acidic proteins that contain a flavin cofactor (flavin mononucleotide, FMN) acting as an electron transfer center [27]. They are small (14.5–23 kDa) α/β proteins with five α-helices packing against a central five-stranded β-sheet, thus forming an αβα sandwich [27,101]. In some bacteria (e.g., *Escherichia coli*, *Azotobacter vinelandii*, or *Desulfovibrio vulgaris*), flavodoxins are constitutive proteins, while in others, such as in several *Anabaena* strains, flavodoxin synthesis is induced in low iron conditions, where it replaces ferredoxin [102,103,104,105,106,107], a constitutive sulfoferric protein that transfers electrons one by one. Although the FMN in flavodoxin only participates in two-electron transfer reactions when it is free in solution, apoflavodoxins modify the redox potentials of FMN molecules bound to them so that they can accept and donate electrons one by one [27]. Flavodoxins transfer electrons among different partner proteins. In some photosynthetic and/or N_2_-fixing bacteria, flavodoxin shuttles electrons from PSI to NADP^+^ or N_2_ via FNR and nitrogenase, respectively [27,108,109]. In an analogous fashion, flavodoxin donates electrons to a variety of partner proteins in different bacteria to perform biosynthetic reactions. For example, flavodoxin activates cobalamin-dependent methionine synthase, pyruvate formate-lyase, and anaerobic ribonucleotide reductase in *Escherichia coli* [110,111,112,113], as well as biotin synthase in *Escherichia coli* [111,112] and *Bacillus subtilis* [114]. Flavodoxin is also involved in nitrate reduction in *Azotobacter vinelandii* [115] and in the activation of pyruvate formate-lyase by free radicals formation [116]. On the other hand, flavodoxin has been found to function as an electron acceptor of the pyruvate oxidoreductase enzyme complex (POR) which catalyzes the oxidative decarboxylation of pyruvate in *Hp* [44] (Figure 2).

**Figure 3:**
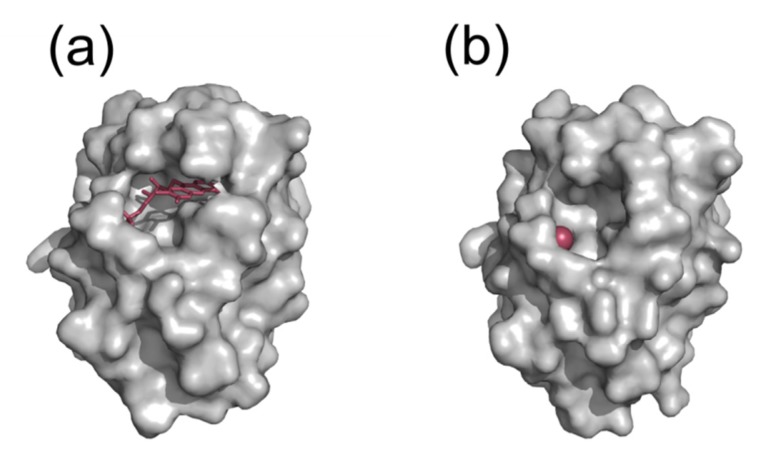
Molecular surface representation of holo (**a**) and apo (**b**) flavodoxin from *Hp*. FMN cofactor and a chloride ion bound at the FMN phosphate site are shown as red sticks and a sphere, respectively. The two structures are similar and exhibit an unusual pocket close to the cofactor binding site. Most other (apo)flavodoxins lack such surface pocket.

The FMN cofactor in flavodoxin is made of an isoalloxazine aromatic ring system connected to a phosphate group by a ribityl chain, and it appears tightly bound at the carboxy-terminal end of the flavodoxin β-sheet. The isoalloxazine moiety usually interacts with aromatic residues, while the phosphate group forms hydrogen bonds with mainly threonine side chains and several main chain NH groups of the protein [27,41]. Those interactions provide high stability to the apoflavodoxin FMN complex, which has a formation that begins by interaction of the isoalloxazin group with the folded apoprotein, followed by docking of the phosphate group [27,117]. The phosphate binding site has been described to be pre-formed in *Anabaena* PCC7119, *Hp* [118], and *Streptococcus pneumoniae* [119] apoflavodoxins, probably due to the presence of bound ions that mimic the phosphate group (Figure 3). From sequence alignment and structural considerations, flavodoxins can be divided into two groups: long-chain (18–23 kDa) (e.g., those in *Anabaena* PCC 7119 or *Hp*) and short-chain flavodoxins (14.5–17 kDa) (e.g., those in *Clostridium beijerincki* or *Desulfovibrio vulgaris*). Long-chain flavodoxins contain an extra 20 residue loop intercalated in the β5-strand of the β-sheet [27,120,121] that does not seem to be relevant for protein stability or folding. Its sequence conservation suggests it may play a functional role [120], and it has been suggested to be responsible for the recognition of FNR and methionine synthase in *E. coli* [122]. Despite their differences, long- and short-chain flavodoxins share a similar three-dimensional structure. In both families, sequence conservation is high at the isoalloxazine binding loops (often referred to as the Y- and W-loops), the phosphate binding loop (P-loop) and, in the long-chain ones, their characteristic long loop. In particular, the phosphate binding site is highly conserved, the consensus sequence being T/S-X-T-G-X-T [27,41,120]. On the other hand, the W- and Y-loops usually contain, respectively, Trp and Tyr residues that are involved in the binding of the isoalloxacin moiety in FMN. Some flavodoxins, nevertheless, contain other residues in those key FMN binding positions (e.g., the tryptophan residue of the W-loop appears replaced by methionine in *Clostridium beijerinckii* flavodoxin, by leucine in *Azobacter vinelandii* or by alanine in *Hp*) [28,41,123].

The flavodoxin from *Hp* (*Hp*-Fld) is involved in a metabolic pathway essential for *Hp* viability: the oxidative decarboxylation of pyruvate by the pyruvate oxidoreductase complex (POR) [27,44,45,124]. Flavodoxin synthesis in *Hp* is constitutive and detectable even in dormant forms of *Hp* which have a significantly reduced metabolic activity [125]. *Hp* flavodoxin has been related to low-grade gastric mucosa-associated lymphoid tissue (MALT) lymphoma pathogenesis, and antibodies against this flavodoxin have been found in patients [126]. In anaerobic conditions, flavodoxin is able to activate imidazole antimicrobials (such as metronidazole) [124,127] and transform them into reactive intermediates that cause DNA lethal damage [124,127,128]. Mutations in ferredoxin (FdxA), ferredoxin-like protein (FdxB), NAD(P)H flavinnitroreductase (FrxA), oxygen-insensitive NAD(P)H nitroreductase (RdxA), flavodoxin (FldA), the γ-subunit of 2-oxoglutarate oxidoreductase (OorD) or the γ-subunit of pyruvate ferredoxin oxidoreductase (PorD) have been related to metronidazole resistance in *Hp* [129,130]. *Hp* flavodoxin is encoded by the *fldA* gene [41,131], and its 164 residue amino acid sequence is similar to that of other flavodoxins, especially long-chain ones (sequence identities approximately 40%). Sequence differences are noticed at the cofactor binding site, specifically at the phosphate loop which, in *Hp*, is slightly different (T-D-S-G-N-A) from the general flavodoxin motif (T/S-X-T-G-X-T) [41]. The *Hp*-Fld structure is similar to that of other known flavodoxins, although the presence of some shorter loops and of an elevated percentage of small side-chain residues makes it slightly more compact [41]. Interestingly, in *Hp* flavodoxin, a bulky residue located in the W-loop of most flavodoxins (typically a Trp residue), appears replaced by an alanine residue (position 55). This substitution, which lowers the affinity for FMN [41], opens a pocket at the protein surface, near the bound cofactor [41,132], where small organic compounds could bind and inhibit complex formation or electron transfer reactions with partner proteins.

As *Hp* lacks several essential genes of the glycolysis pathway, pyruvate formation through carbon dioxide fixation may be physiologically favored, because it is the single gluconeogenic pathway in this bacterium [46]. On the other hand, oxidative decarboxylation of pyruvate is a fundamental reaction catalyzed by the pyruvate dehydrogenase complex in most aerobic organisms or by POR in anaerobic ones [133,134]. Thus, *Hp* POR catalyzes the last step of carbohydrates fermentation as well as the inverse pyruvate oxidative decarboxylation [135]. In this bidirectional electron transfer pathway, another essential enzyme, flavodoxin quinone reductase B (Figure 2), is involved [46].

## 4. Flavodoxins in other Pathogenic Bacteria and in the Gut Microbiota

As flavodoxin is present in gastrointestinal pathogens other than *Hp*, the potential of flavodoxin inhibitor-based therapies against those additional pathogens should be explored, especially in the cases of pathogens for which an essential flavodoxin has been described. On the other hand, flavodoxin is also present in a variety of gut commensal bacteria and the possible negative side effects of *Hp* flavodoxin inhibitors on the human microbiota should be evaluated too.

Indeed, flavodoxin genes are present in many bacteria (mainly Gram-negative ones), especially in Proteobacteria, Cyanobacteria, Aquificae, Firmicutes, Bacteroidetes, Fusobacteria, and Spirochaetes. We have collected and combined in Table 2 flavodoxin information available in the Uniprot database [136] (searching for “flavodoxin” and refining by “reviewed”), in the NCBI database [137] (searching for “flavodoxin” on the “Protein” tab, then refining by “Bacteria” on the species tag, selecting on the source databases’ tag “PDB” and “UniProtKB/Swiss-Prot”, and specifying on the sequence length’s tag from 130 to 199 residues), and in the flavodoxin-related literature available in PubMed [137].

Most of these flavodoxins appear in Proteobacteria, and some of them are known to be essential for bacterial viability. Essential flavodoxins annotated in the DEG database [160] and retrieved by searching for “flavodoxin” by function in the bacteria’s tab are shown in Table 3.

**Figure A1:**
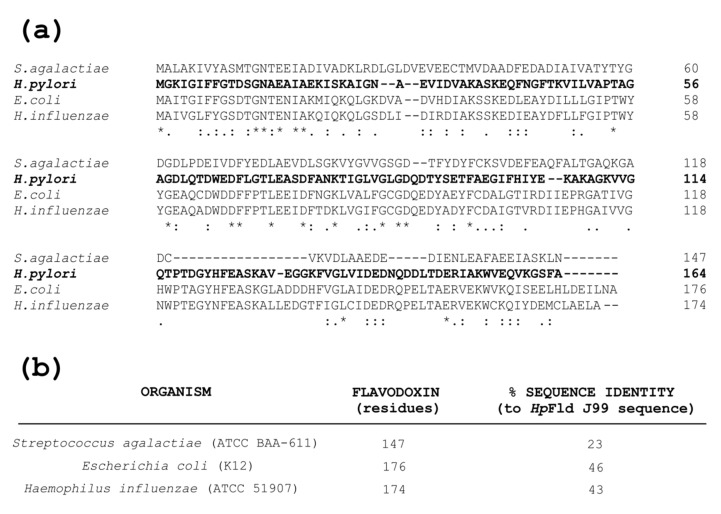
(**a**) Multiple sequence alignment of flavodoxins from *Streptococcus agalactiae* (ATCC BAA-611), *Hp* (strain J99), *Escherichia coli* (strain K12), and *Haemophilus influenzae* (strain ATCC 51907). It has been performed with Clustal Omega [194]. Asterisks (*) indicate positions with a single residue; colons (:) indicate conservation between groups of strongly similar properties; dots (.) indicate conservation between groups of weakly similar properties. (**b**) Sequence identity between *Hp* (strain J99) flavodoxin and that from *Streptococcus agalactiae* (ATCC BAA-611), *Escherichia coli* (strain K12), and *Haemophilus influenzae* (strain ATCC 51907). It has been calculated with Clustal Omega [194].

To anticipate the potential side effects on the human gastric microbiota associated to the use of flavodoxin-targeting therapies, we have revised the presence of flavodoxin in these commensal bacteria. Some of the key organisms present in the human gastric microbiota are shown in Table 4 classified by genus, phylum, gram staining, oxygen requirement, and (non)presence of flavodoxin(s). Essential flavodoxins are indicated, where appropriate. As shown in the table, a variety of bacterial genera from the human gastric microbiota have been identified as flavodoxin-expressing organisms. Particular care should be taken with these microorganisms in flavodoxin-based treatments, especially with *Escherichiacoli*, *Haemophilus influenzae*, and *Streptococcus agalactiae* which have flavodoxins that are essential for bacterial viability. Besides, the flavodoxins from the first two ones share a high percentage of identity (above 40%) with *Hp*-Fld (Figure A1, Appendix A). As sequence identity levels higher than 35–40% usually involve substantial structural similarity [163], it is important to ensure that compounds developed against *Hp* flavodoxin are not able to kill these microorganisms at the minimal inhibitory concentrations (MIC) determined for *Hp*. On the other hand, it has been proposed that flavodoxin-inhibitors could interact with the protein near its cofactor binding site and then suppress flavodoxin function by modification of the cofactor redox potential or by steric blockage of the interaction between the flavodoxin and its redox partners [28]. Therefore, if *Hp*-Fld inhibitors bind the protein at the pocket created by the presence of an alanine at position 55, no side effects on the human microbiota would be expected because *E. coli* and *H. influenzae* flavodoxins have a bulky tryptophan residue at this position and that of *S. agalactiae* contains an also bulky tyrosine residue. (Figure A1, Appendix A)

**Figure A2:**
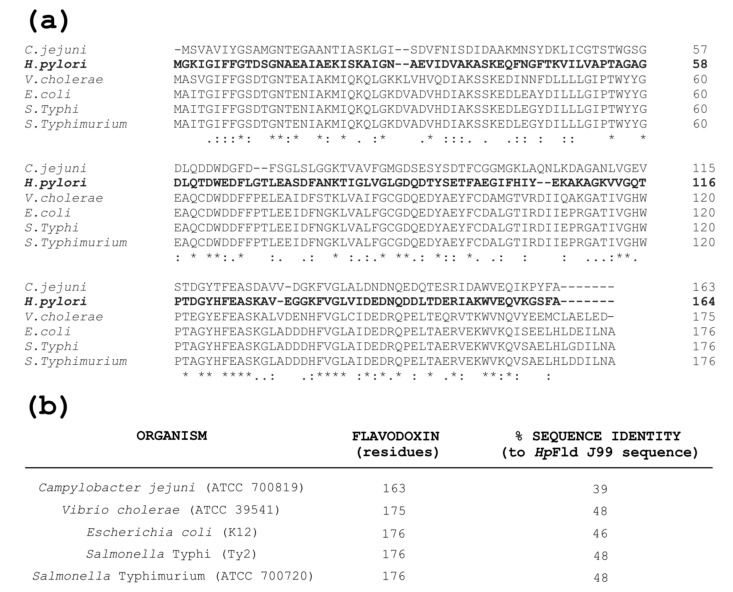
(**a**) Multiple sequence alignment of flavodoxins from *Campylobacter jejuni* (strain ATCC 700819), *Hp* (strain J99), *Vibrio cholerae* (strain ATCC 39541), *Escherichia coli* (strain K12), *Salmonella enterica* subsp. *enterica* serovar Typhi (strain Ty2) and *Salmonella enterica* subsp. *enterica* serovar Typhimurium (strain ATCC 700720). It has been performed with Clustal Omega [194]. (**b**) Sequence identity between *Hp* (strain J99) flavodoxin and that from *Campylobacter jejuni* (strain ATCC 700819), *Vibrio cholerae* (strain ATCC 39541), *Escherichia coli* (strain K12), *Salmonella enterica* subsp. *enterica* serovar Typhi (strain Ty2), and *Salmonellaenterica* subsp. *enterica* serovar Typhimurium (strain ATCC 700720). It has been calculated with Clustal Omega [194].

On the other hand, flavodoxin is present in gastrointestinal pathogens, for some of which it is an essential protein. In Table 5, major human stomach pathogens are classified by genus, phylum, gram staining, oxygen requirement, and flavodoxin expression (Yes/No). If flavodoxin is essential for their viability, it is indicated. While different bacteria included in this table (Firmicutes, Bacteroidetes, and Proteobacteria) express flavodoxin, it has been described so far as essential only for some Proteobacteria: *Campylobacter, Escherichia, Helicobacter, Salmonella*, and *Vibrio*. As shown in Figure A2 (Appendix A), sequence identities between *Hp* (strain J99) flavodoxin and those from *Campylobacter jejuni* (strain ATCC 700819), *Vibrio cholerae* (strain ATCC 39541), *Escherichia coli* (strain K12), *Salmonella enterica* subsp. *enterica* serovar Typhi (strain Ty2), and *Salmonellaenterica* subsp. *enterica* serovar Typhimurium (strain ATCC 700720) are around 40% which is high enough to assume these proteins will show a high structural similarity [163]. Nevertheless, the amino acid sequences of these five flavodoxins, having a tryptophan residue where *Hp*-Fld carries an alanine one, suggest their tridimensional structures will not display a pocket near the FMN cofactor, and they may not be affected by *Hp*-Fld inhibitors.

## 5. Discovery of Specific Inhibitors of *Hp* Flavodoxin Using an Approach that Can Be Transferred to Other Pathogens

The eradication rates achieved by the conventional triple and quadruple therapies used for treating *Hp* infection have been decreasing, so alternative treatments are needed to fight this prevailing infection. Among the different strategies available, development of inhibitors of essential *Hp* targets seems promising. Following this track, our group has focused on the identification and improvement of small molecule inhibitors to target the essential *Hp* flavodoxin. As explained above, *Hp* flavodoxin bears in its surface a peculiar pocket near the FMN binding site that could be exploited to identify molecules that bind there and block function by either modifying the redox potential of the protein or by sterically interfering with the recognition of partner proteins [28]. The *Hp*-Fld surface pocket is absent in other epsilonproteobacteria, such as *Campylobacter jejuni* or *Wolinella succinogenes*, that carry the characteristic Trp residue of the W-loop, or in other flavodoxins where the Trp residue has been replaced by leucine (e.g., in *Azotobacter vinelandii* [123]) or methionine (e.g., in *Clostridium beijerinkii* [41]). The pocket is not expected either to be present in several *Helicobacter* species such as *Helicobacter hepaticus* (unable to colonize the human gastric mucosa but associated with chronic hepatitis, liver adenocarcinoma in mice, cholecystitis and gallbladder human cancer) [184,185], where its flavodoxin carries a tyrosine replacing the Trp. However, the pocket probably appears in the flavodoxins from *Helicobacter acinonychis* (a bacteria establishing lifelong infections in the stomach of cheetah and other felines) [186] and *Helicobacter felis* (which has been related to the development of gastritis in humans) [187] as, similarly to *Hp* flavodoxin, they carry an alanine residue replacing the Trp. Thus, the pocket initially observed in *Hp*-Fld as formed by replacement of the bulky Trp residue at the W-loop by an Ala residue is an almost exclusive feature of *Hp*-Fld which can be used to develop highly selective inhibitors. A priori, such new inhibitors would not give rise to side effects in humans, because flavodoxin is not present in vertebrates. An alternative possibility to use flavodoxin as an antimicrobial target stems from the fact that redox proteins have been suggested to be capable of activating nitro-compounds, as it has been demonstrated for nitroimidazole drugs such as metronidazole. Reduction of nitro groups present in prodrugs could yield cytotoxic products which would be able to act as anti-*Hp* compounds [124,127,128,129,188,189,190,191,192].

**Figure 4:**
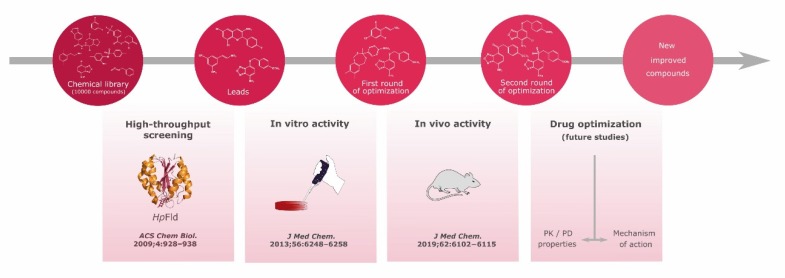
The steps followed and planned in the discovery of flavodoxin inhibitors as new therapies against *Hp* infection.

Our research group has been working on the identification and development of new compounds targeting *Hp* flavodoxin by following the alternative possibilities of blocking flavodoxin function using binding molecules and that of using compounds that could be activated by their conversion into reactive toxic species after reduction (Figure 4). First, small molecules, such as benzylamine, were identified as binders to *Hp*-Fld [28]. Due to the fact of their low binding affinity, they were poor inhibitors. A high-throughput screening method was subsequently implemented to identify them from a chemical library of other small organic molecules which could bind tighter to the protein. Thus, a 10,000 molecule chemical library was screened using pure recombinant *Hp*-Fld which led to the identification of 29 binding compounds that stabilized the protein as indicated by their capability to increase the temperature of mid-denaturation [42]. Four of those compounds (compounds I, II, III, and IV) were, in addition, able to inhibit the in vitro electron transfer between Fld and their recombinantly produced partner proteins POR and FqrB. Three of them (I, II, and IV) showed bactericidal activity against *Hp* cells and seemed to be selective for this bacterium. Inhibitors I and II exhibited therapeutic indexes (TI) of around 10, meaning their minimal cytotoxic concentrations (MCC) for eukaryotic cells were 10 times higher than the corresponding minimal inhibitory concentrations (MIC) for *Hp*. Nonetheless, compound IV showed lower TI due to the higher cytotoxicity and lower efficacy than molecules I and II. On the other hand, those three compounds (I, II, and IV) did not seem to be nephrotoxic or hepatotoxic when they were administered to mice at 10 mg/kg body weight, and they did not produce pathological changes in stomach, liver, heart, lung or kidney at 1 or 10 mg/kg body weight. In an attempt to obtain detailed structural information on the complexes formed by the inhibitors with the target, the interaction of compounds III (the only bacteriostatic compound of the four hits) and IV with *Hp*-Fld was studied by crystallography and NMR, respectively. Inhibitor III was able to replace FMN and establish hydrogen bonds and hydrophobic interactions with the protein through its nitro group and benzene ring, respectively. However, it was unclear whether the structure solved was showing the functional inhibitory interaction or rather the FMN replacement by the inhibitor occurred in a subsequent step. The NMR analysis indicated that compound IV also appeared to interact with flavodoxin through its nitro group which suggested a role for this functional group in the formation of the inhibitor–flavodoxin complex. Crystallization trials are underway to try to obtain the X-ray structure of the complex between flavodoxin and compound IV and derivatives of it.

In a first round of optimization of the initial inhibitors, 102 new molecules related to inhibitors I, II, and IV were synthetized or acquired, and their toxicity and activity were tested [43]. Among them, 20 compounds were able to bind to flavodoxin with dissociation constants in the micromolar range, some of them with higher affinity than those of the initial hits. Most of these analogues inhibited bacterial growth in vitro and nine of them showed higher therapeutic indexes than those of their parent bactericidal compounds I, II, and IV. Large increases were observed in the therapeutic indexes of the new analogues of II and IV (up to 25 times for derivatives of II and up to 59 times for derivatives of IV). Six of these compounds were further tested, and it was confirmed that they kept a similar binding affinity to flavodoxin and that they also displayed bactericidal properties [43].

A second round of optimization was then carried out. To improve the therapeutic and pharmacokinetic properties of compounds I, II, and IV, new variants carrying modified redox forms of nitro, sulfur and vinyl groups of the lead-molecules were synthesized [193]. Derivatives that contain partially or fully reduced forms of the nitro and/or ethylene groups, or partially or fully oxidized forms of the sulfur atom displayed a considerably lower toxicity against HeLa cells and mice than the corresponding leads. While the therapeutic indexes of derivatives of I or II did not represent a significant improvement, some of the derivatives of IV were effective, according to EUCAST (The European Committee on Antimicrobial Susceptibility Testing) criteria, against *Hp* clinical isolates resistant to common antibiotics such as metronidazole, clarithromycin, and rifampicin. Furthermore, four of these new compound IV derivatives, used as sole agents, were able to significantly reduce *Hp* gastric colonization in the mouse model of infection and indeed to eradicate the infection in some mice. At present, we will continue the development of derivatives of compound IV through the design and testing of more soluble variants with optimized metabolic stability and bioavailability, and we are testing their effect on other bacteria including those present in the gastric microbiota.

Our preliminary results indicate the *Hp*-Fld inhibitors so far developed are highly specific for this bacterium, and that they do not show activity (or very low) against a representative panel of other bacteria from different phyla. This selectivity will be useful to minimize the generation of resistances and suggests these inhibitors will be less damaging to the gut microbiota than broad-spectrum antibiotics. Thus, this new family of selective *Hp*-inhibitors could provide an opportunity for the formulation of therapeutic alternatives to fight *Hp*-drug resistant strains [193]. As explained, due to the fact of their high selectivity, these *Hp*-inhibitors will not likely be effective against other pathogens. However, other bacterium-specific flavodoxin inhibitors can be identified anew, though specific screening of chemical libraries against other essential flavodoxins. Such bacterium-specific flavodoxin-inhibitors could also be developed into novel antimicrobials against gastrointestinal pathogens such as *Bacillus*, *Campylobacter*, *Listeria*, *Salmonella*, *Shigella* or *Vibrio*. To do so, the target-based approach followed to discover *Hp*-Fld inhibitors can be readily applied to identify specific antimicrobials against other flavodoxin-containing pathogens. Moreover, the steps carried out to improve the therapeutic and pharmacokinetic properties of the *Hp*-Fld inhibitors could also be followed in order to enhance the antimicrobial properties of these compounds against those bacteria.

## Figures and Tables

**Table 1:** Targets for *Hp* infection treatment.

Target	Pathway	Reference
**Metabolism**		
Type II 3-dehydroquinase dehydratase (DHQ2)	Shikimate pathway	[30,31,32,33]
Shikimate 5-dehydrogenase (SDHase)	Shikimate pathway	[30,33]
Shikimate kinase (SK)	Shikimate pathway	[30,33,34]
Chorismate synthase	Shikimate pathway	[30,33]
Phosphopantetheine adenylyltransferase (PPAT)	Coenzyme A biosynthesis	[35,36]
Carbon starvation protein A	Starvation response, utilization of peptides, and host–pathogen interactions	[37]
Methylthiotransferase (MiaB)	Protein synthesis	[37]
Ribosomal RNA small subunit methyltransferase E	Protein synthesis	[37]
Ribosomal protein L11 methyltransferase	Protein synthesis	[37]
Tetrapyrrole (Corrin-Porphyrin) methylase family protein	Protein synthesis	[37]
Peptide chain release factor 1	Protein synthesis	[37]
Fumarate reductase (FrdA, FrdB, and FrdC)	Krebs cycle and anaerobic respiration	[33,38]
Glu-tRNA^Gln^ amidotransferase, subunits A (GatA), B (GatB), and C (GatC)	Protein synthesis	[26,33]
Helicase-nuclease DNA Repair Enzymes (AddAB)	DNA damage reparation	[39,40]
Cytochrome C-type biogenesis protein CcdA	Cytochrome C synthesis	[37]
Cytochrome C oxidase, subunits CcoN, CcoO, CcoP and CcoQ	ATP synthesis	[37]
Flavodoxin (Fld)	Oxidative decarboxylation of pyruvate	[28,41,42,43]
Pyruvate:ferredoxin oxidoreductase (POR), subunit α (porA), β (porB), ϒ (porC or porG) and δ (porD)	Oxidative decarboxylation of pyruvate	[26,33,44,45,46]
Flavodoxin:quinone reductase (FqrB)	Oxidative decarboxylation of pyruvate	[33,42,46]
2-oxoglutarate:acceptor oxidoreductase, subunits A (OorA), B (OorB), C (OorC) and D (OorD)	Succinyl-CoA production	[26,33,45]
**Cell Wall Structure**		
N-succinyl-L,L-diaminopimelic acid desuccinylase, SDAP-deacylase (DapE)	Succinylase pathway (lysine biosynthesis)	[17,47,48]
Glutamate racemase MurI	Peptidoglycan biosynthesis	[49,50]
Multi-drug resistance protein MsbA	Lipopolysaccharide biosynthesis	[51]
UDP-galactose 4-epimerase (GalE)	Lipopolysaccharide biosynthesis	[52]
**pH Homeostasis**		
Urease, subunits α (UreA), and β (UreB)	Acclimation to low pH	[33,53,54,55,56,57,58]
Nickel-responsive regulator (NikR)	Urease expression and nickel uptake regulator	[53,54]
Nickel–cobalt transporter (NixA)	Nickel/cobalt transport	[54,59,60]
Urease accessory protein UreE	Urease maturation	[33,54,59,61,62,63]
Urease accessory protein UreF	Urease maturation	[54,61,62]
Urease accessory protein UreG	Urease maturation	[33,54,62,63]
Urease accessory protein UreH	Urease maturation	[54,61]
Hydrogenase/urease maturation factor (HypA)	Urease maturation	[61,63]
Hydrogenase/urease maturation factor (HypB)	Urease maturation	[61,63]
Heat Shock Protein A (HspA)	Nickel homeostasis	[59,64,65]
Hpn	Nickel homeostasis and storage	[59,61,64]
Acid-activated urea channel (UreI)	Urea permeability	[33,66]
α-carbonic anhydrase	Acclimation to low pH	[33,67,68,69,70,71]
β-carbonic anhydrase	Acclimation to low pH	[33,67,68,69,71,72]
**Virulence (Adherence, Motility and Pathogenicity)**		
Spore coat polysaccharide biosynthesis protein C (PseC)	Pseudaminic acid biosynthesis pathway (Pse): flagellin glycosylation	[33,73,74]
Heat-inducible transcription repressor (HrcA)	Flagella biosynthesis	[37]
Transcriptional repressor of DnaK operon (HspR)	Flagella biosynthesis	[37]
Major flagellin FlaA	Flagellar filament composition	[33,53,75,76,77]
Minor flagellin FlaB	Flagellar filament composition	[53,75,76,77]
Flagellar hook-associated protein 2 (FliD)	Flagellum assembly (filament capping)	[33,76,77]
Flagellar hook-associated protein 1 (FlgK)	Flagellum assembly (hook-filament junction formation)	[33,76,77,78]
ATP-binding protein (YlxH)	Flagella biosynthesis	[33,79]
Flagellar basal body L-ring protein (FlgH)	Flagellum assembly (L-ring composition)	[33,77]
Flagellar basal body P-ring protein (FlgI)	Flagellum assembly (P-ring composition)	[33,77]
Flagellar basal body M-ring protein (FliF)	Flagellum assembly (MS ring composition)	[33,77]
Flagellar biosynthetic protein (FliP)	Flagellum assembly (Flagellar export component)	[33,60,77]
Flagellar biosynthetic protein (FliQ)	Flagellum assembly (Flagellar export component)	[33,77]
Flagellar motor switch protein (FliY)	Flagellum assembly (C-ring composition; Flagellar export component)	[33,77]
Flagellum-specific ATP synthase (FliI)	Flagellum assembly (Flagellar export component)	[33,77]
Flagella-specific σ factor (FliA)	Flagellum assembly (regulatory protein)	[53,77]
FlgM (putative antagonist of FliA)	Flagellum assembly (regulatory protein)	[53,77]
Cytotoxin-associated gene A (CagA)	*cag* pathogenicity island (host cell metabolism modulation, inflammation, metaplasia and precancerous transformations)	[80,81,82,83]
*cag*-Type IV secretion system (T4SS)	*cag* pathogenicity island (translocation of bacterial factors (e.g., Cag A and peptidoglycan) into host cells)	[80,83]
HopQ adhesin (outer membrane protein)	Adhesion to host cells and translocation of CagA into host cells	[84]
Vacuolating cytotoxin (VacA)	Cellular vacuolation, apoptosis and inhibition of cell cycle progression and host immune response	[81,82,85,86]
Blood group antigen binding adhesin (BabA)	Adhesion to host cells	[80,81,82]
High temperature requirement A (HtrA)	Chaperone and proteolytic activities (intercellular adhesion cleavage)	[37,85,87]
Sialic acid-binding adhesin (SabA) (outer membrane protein)	Bacterial migration to epithelium surface	[76,88]
HopZ adhesin (outer membrane protein)	Adhesion to host cells	[76,80,85]
OipA adhesin (outer membrane protein)	Adhesion to host cells	[76,80,85]
AlpA/B adhesin (outer membrane protein)	Adhesion to host cells	[76,80,85]
**Active Efflux of Metal Ions**		
Cation efflux system protein CusA	Efflux of cobalt/zinc/cadmium	[37]
Cobalt/Zinc/Cadmium efflux system membrane fusion protein	Efflux of cobalt/zinc/cadmium	[37]
Cobalt/Zinc/Cadmium resistance protein (CzcA, CzcB and CzcC)	Efflux of cobalt/zinc/cadmium	[37,89]
CznABC metal efflux pump	Efflux of cadmium/zinc/nickel	[89]
Ferrix siderophore transport system TonB periplasmic binding protein	Iron transport	[37]
Ferric siderophore transport system ExbB biopolymer transport protein	Iron transport	[37,60]
Haemin uptake system ATP binding protein	Iron transport	[37]
**Protection Against Stress**		
Glutathionyl spermidine synthetase	Intracellular thiol redox balance regulation	[37]
Iron-binding ferritin-like antioxidant protein	Prevention of toxic reactive species formation	[37]
DNA-binding protein Dps	DNA breaking protection	[37]
Superoxide dismutase	Superoxide dismutation	[37]
Thioredoxin reductase	Prevention of toxic reactive species formation	[37]
RNA polymerase σ^54^ factor	Survival under stress conditions	[33,90]
Multi-drug resistance protein MsbA	Efflux of hydrophobic drugs	[33,51]
Exodeoxyribonuclease (LexA)	SOS response activation	[33,91]
Homeostatic stress regulator (HsrA)	Regulation of gene expression	[92,93,94,95]

**Table 2:** Some flavodoxin-containing bacteria ^a^.

Microorganism	Protein Name	Gene Name	Seq. Length	Long/short Chain	Phylum	Gram Stain
*Anabaena (Nostoc*) sp.	Flavodoxin	*isiB*	170	Long	Cyanobacteria	Negative
*Aquifex aeolicus*	Flavodoxin	*fldA*	185	Long	Aquificae	Negative
*Azotobacter vinelandii*	Flavodoxin 1	*Avin45950*	174	Long	Proteobacteria	Negative
*Azotobacter vinelandii*	Flavodoxin 2	*nifF*	180	Long	Proteobacteria	Negative
*Azotobacter chroococcum*	Flavodoxin B	*nifF*	180	Long	Proteobacteria	Negative
*Bacillus cereus*	Flavodoxin	*BC_1376*	148	Short	Firmicutes	Positive
*Bacillus cereus*	Flavodoxin	*BC_3541*	154	Short	Firmicutes	Positive
*Bacillus subtilis*	Probable flavodoxin 2	*ykuP*	151	Short	Firmicutes	Positive
*Bacillus subtilis*	Probable flavodoxin 1	*ykuN*	158	Short	Firmicutes	Positive
*Bacteroides uniformis*	Flavodoxin	*BACUNI_04544*	178	Long	Bacteroidetes	Negative
*Buchnera aphidicola*	Flavodoxin	*fldA BUsg_289_*	154	Long ^b^	Proteobacteria	Negative
*Buchnera aphidicola*	Flavodoxin	*fldA BU299*	171	Long	Proteobacteria	Negative
*Buchnera aphidicola*	Flavodoxin	*fldA bbp_277*	174	Long	Proteobacteria	Negative
*Campylobacter jejuni*	Flavodoxin	*fldA*	163	Long	Proteobacteria	Negative
*Clostridium beijerinckii* ^c^	Flavodoxin		138	Short	Firmicutes	Positive
*Clostridium pasteurianum*	Flavodoxin	*CLPA_c13840* *^d^*	140	Short	Firmicutes	Positive
*Clostridium saccharobutylicum*	Flavodoxin	*floX*	160	Long	Firmicutes	Positive
*Desulfovibrio desulfuricans*	Flavodoxin	*Ddes_1951*	148	Short	Proteobacteria	Negative
*Desulfovibrio gigas* ^c^	Flavodoxin		146	Short	Proteobacteria	Negative
*Desulfovibrio gigas* ^c^	Flavodoxin		147	Short	Proteobacteria	Negative
*Desulfovibrio salexigens*	Flavodoxin	*Desal_0805*	146	Short	Proteobacteria	Negative
*Desulfovibrio vulgaris*	Flavodoxin	*DVU_2680/DvMF_1143*	148	Short	Proteobacteria	Negative
*Escherichia coli*	Protein MioC	*mioC*	147	Short	Proteobacteria	Negative
*Escherichia coli*	Uncharacterized protein YqcA	*yqcA*	149	Short	Proteobacteria	Negative
*Escherichia coli*	Flavodoxin 2	*fldB*	173	Long	Proteobacteria	Negative
*Escherichia coli*	Flavodoxin 1	*fldA*	176	Long	Proteobacteria	Negative
*Fusobacterium nucleatum*	Flavodoxin	*FN0724*	167	Long	Fusobacteria	Negative
*Haemophilus influenzae*	Protein MioC homolog	*mioC*	146	Short	Proteobacteria	Negative
*Haemophilus influenzae*	Flavodoxin	*fldA*	174	Long	Proteobacteria	Negative
*Helicobacter pylori*	Flavodoxin	*fldA*	164	Long	Proteobacteria	Negative
*Klebsiella pneumoniae*	Flavodoxin	*fldA/nifF*	176	Long	Proteobacteria	Negative
*Lactobacillus reuteri*	Flavodoxin/nitric oxide synthase	*Lreu_1727*	149	Short	Firmicutes	Positive
*Listeria monocytogenes*	Lmo2153 protein	*lmo2153*	145	Short	Firmicutes	Positive
*Megasphaera elsdenii*(*Peptostreptococcus elsdenii*) ^c^	Flavodoxin		137	Short	Firmicutes	Negative
*Pantoea agglomerans* (*Enterobacter agglomerans*)	Flavodoxin	*nifF*	177	Long	Proteobacteria	Negative
*Pasteurella multocida*	Protein mioC homolog	*mioC*	147	Short	Proteobacteria	Negative
*Pectobacterium carotovorum* ^c^	Exoenzyme regulation regulon ORF2		151	Short	Proteobacteria	Negative
*Pseudomonas aeruginosa*	Uncharacterized protein PA3435	*PA3435*	150	Short	Proteobacteria	Negative
*Pseudomonas aeruginosa*	Flavodoxin FldP	*fldP*	184	Long	Proteobacteria	Negative
*Pseudomonas putida*	Flavodoxin	*mioC*	151	Short	Proteobacteria	Negative
*Rhodobacter capsulatus*	Flavodoxin	*nifF*	182	Long	Proteobacteria	Negative
*Salmonella* Typhi	Flavodoxin 2	*fldB*	173	Long	Proteobacteria	Negative
*Salmonella* Typhi	Flavodoxin	*fldA*	176	Long	Proteobacteria	Negative
*Salmonella* Typhimurium	Flavodoxin 2	*fldB*	173	Long	Proteobacteria	Negative
*Salmonella* Typhimurium	Flavodoxin 1	*fldA*	176	Long	Proteobacteria	Negative
*Shewanella oneidensis*	Flavodoxin Protein MioC	*mioC*	146	Short	Proteobacteria	Negative
*Shewanella oneidensis*	tRNA pseudouridine synthase C-associated flavoprotein YqcA	*yqcA*	154	Short	Proteobacteria	Negative
*Shewanella oneidensis*	Flavodoxin	*fldA*	175	Long	Proteobacteria	Negative
*Shigella flexneri*	Uncharacterized protein YqcA	*yqcA*	149	Short	Proteobacteria	Negative
*Shigella flexneri*	Flavodoxin 1	*fldA*	176	Long	Proteobacteria	Negative
*Streptococcus agalactiae ^e^*	Flavodoxin	*mioC*	147	Short	Firmicutes	Positive
*Streptococcus pneumoniae*	Flavodoxin	*flaV*	147	Short	Firmicutes	Positive
*Synechococcus sp.*	Flavodoxin	*isiB*	170	Long	Cyanobacteria	Negative
*Synechocystis sp.*	Flavodoxin	*isiB*	170	Long	Cyanobacteria	Negative
*Treponema pallidum*	Flavodoxin	*fldA*	146	Short	Spirochaetes	Negative ^f^
*Trichodesmium erythraeum*	Flavodoxin	*fld*	171	Long	Cyanobacteria	Negative
*Vibrio cholerae*	Protein MioC homolog	*mioC*	144	Short	Proteobacteria	Negative
*Vibrio cholerae*	Flavodoxin	*fld1*	175	Long	Proteobacteria	Negative
*Vibrio cholerae*	Flavodoxin	*fld2*	198	Long	Proteobacteria	Negative
*Wolinella succinogenes*	Flavodoxin	*fldA*	171	Long	Proteobacteria	Negative

^a^ Extracted from Uniprot by searching for “flavodoxin” and refining by “reviewed”, from NCBI by searching for “flavodoxin” in the “Protein” tab and refining by “Bacteria” (in the species tag), “PDB and UniProtKB/Swiss-Prot” (in the source databases’ tag) and “from 130 to 199 residues” (in the sequence length’s tag) and from References [114,119,138,139,140,141,142,143,144,145,146,147,148,149,150,151,152,153,154,155,156,157,158,159]. Despite the fact that there is a great deal of unreviewed flavodoxin sequences in Uniprot, we chose to include only those that we found as described, which were flavodoxins with an existence that appeared to be firmly established. ^b^ Although the length of this sequence is more typical of short-chain flavodoxins, we classified it here as long-chain due to the absence, in sequence alignment with long chain-flavodoxins, of the characteristic 20 residue gap formed in so-aligned short-chain sequences. ^c^ Unnamed gene (gene name not reported yet. Alternative sequences may be reported elsewhere). ^d^ Although the isolation of a 148 residue flavodoxin from *Clostridium pasteurianum* has been reported [157,159], we did not find any sequences of such length in Uniprot. On the other hand, the sequence reported in those papers was not complete. Among the sequences in Uniprot, the one which is 140 residues in length (gene name *CLPA_c13840*) has the highest identity in sequence with the partial sequences reported. ^e^ Extracted from the DEG database [160]. This sequence was not identified by following the search pathway used for the rest of the sequences reported in the table. ^f^ Its Gram stain classification has been controversial [161,162].

**Table 3:** Bacteria with flavodoxins that are essential for viability ^a^.

Microorganism	Sequence Length	Long/Short Chain	Phylum	Gram Stain
*Campylobacter jejuni*	163	Long	Proteobacteria	Negative
*Escherichia coli*	176	Long	Proteobacteria	Negative
*Haemophilus influenzae*	174	Long	Proteobacteria	Negative
*Helicobacter pylori*	164	Long	Proteobacteria	Negative
*Salmonella* Typhi	176	Long	Proteobacteria	Negative
*Salmonella* Typhimurium	176	Long	Proteobacteria	Negative
*Shewanella oneidensis*	175	Long	Proteobacteria	Negative
*Streptococcus agalactiae*	147	Short	Firmicutes	Positive
*Vibrio cholerae*	175	Long	Proteobacteria	Negative

^a^ Obtained from DEG database [160].

**Table 4:** Flavodoxin in the main bacterial genera of the human gut microbiota ^a^.

Genus	Flavodoxin	Phylum	Gram Stain	Oxygen Requirement
*Akkermansia*	Unreviewed	Verrucomicrobia	Negative	Anaerobe
*Alistipes*	Unreviewed	Bacteroidetes	Negative	Anaerobe
*Bacteriodes*	Yes	Bacteroidetes	Negative	Anaerobe
*Bifidobacterium*	Unreviewed	Actinobacteria	Positive	Anaerobe
*Clostridium*	Yes	Firmicutes	Positive	Anaerobe
*Eggerthella*	Unreviewed	Actinobacteria	Positive	Anaerobe
*Enterococcus*	Unreviewed	Firmicutes	Positive	Facultative anaerobe
*Escherichia*	Yes ^b^	Proteobacteria	Negative	Facultative anaerobe
*Eubacterium*	Unreviewed	Firmicutes	Positive	Anaerobe
*Fusobacterium*	Yes	Fusobacteria	Negative	Anaerobe
*Haemophilus*	Yes ^b^	Proteobacteria	Negative	Facultative anaerobe
*Lactobacillus*	Yes	Firmicutes	Positive	Microaerophile
*Neisseria*	No	Proteobacteria	Negative	Aerobe
*Odoribacter*	Unreviewed	Bacteroidetes	Negative	Anaerobe
*Parabacteroides*	Unreviewed	Bacteroidetes	Negative	Anaerobe
*Peptococcus*	Unreviewed	Firmicutes	Positive	Anaerobe
*Peptostreptococcus*	Yes	Firmicutes	Positive	Anaerobe
*Porphyromonas*	Unreviewed	Bacteroidetes	Negative	Anaerobe
*Prevotella*	Unreviewed	Bacteroidetes	Negative	Anaerobe
*Propionibacterium*	Unreviewed	Actinobacteria	Positive	Anaerobe
*Pseudomonas*	Yes	Proteobacteria	Negative	Aerobe
*Roseburia*	Unreviewed	Firmicutes	Positive	Anaerobe
*Rothia*	Unreviewed	Actinobacteria	Positive	Anaerobe
*Ruminococcus*	Unreviewed	Firmicutes	Positive	Anaerobe
*Staphylococcus*	Unreviewed	Firmicutes	Positive	Facultative anaerobe
*Streptococcus*	Yes ^b^	Firmicutes	Positive	Facultative anaerobe
*Veillonella*	Unreviewed	Firmicutes	Negative	Anaerobe

^a^ The information related to the bacterial composition of the human gut microbiota was extracted from References [164,165,166,167,168,169,170]. Unreviewed indicates the existence of flavodoxin sequences reported as such in Uniprot. No scientific literature about them has been found. ^b^ Essential flavodoxin according to the DEG database [160].

**Table 5:** Flavodoxin in human gastrointestinal pathogens ^a^.

Genus	Flavodoxin	Phylum	Gram Stain	Oxygen Requirement
*Bacillus*	Yes	Firmicutes	Positive	Aerobe
*Bacteroides*	Yes	Bacteroidetes	Negative	Anaerobe
*Campylobacter*	Yes ^b^	Proteobacteria	Negative	Microaerophile
*Clostridium*	Yes	Firmicutes	Positive	Anaerobe
*Escherichia*	Yes ^b^	Proteobacteria	Negative	Facultative anaerobe
*Helicobacter*	Yes ^b^	Proteobacteria	Negative	Microaerophile
*Listeria*	Yes	Firmicutes	Positive	Facultative anaerobe
*Peptostreptococcus*	Yes	Firmicutes	Positive	Anaerobe
*Salmonella*	Yes ^b^	Proteobacteria	Negative	Facultative anaerobe
*Shigella*	Yes	Proteobacteria	Negative	Facultative anaerobe
*Staphylococcus*	Unreviewed	Firmicutes	Positive	Facultative anaerobe
*Vibrio*	Yes ^b^	Proteobacteria	Negative	Facultative anaerobe
*Yersinia*	Unreviewed	Proteobacteria	Negative	Facultative anaerobe

^a^ The information related to the bacterial genera which cause gastrointestinal diseases was extracted from References [171,172,173,174,175,176,177,178,179,180,181,182,183]. Unreviewed indicates flavodoxin sequences reported as such in Uniprot. No scientific literature about them was found. ^b^ Essential flavodoxin according to the DEG database [160].

## Abbreviations

Ala (A)	Alanine
ATCC	American Type Culture Collection
DNA	Deoxyribonucleic acid
EUCAST	European Committee on Antimicrobial Susceptibility Testing
Fld	Flavodoxin
FMN	Flavinmononucleotide
FqrB	Flavodoxin:quinone reductase
*Hp*	*Helicobacter pylori*
MALT	Gastric mucosa-associated lymphoid tissue lymphoma
MCC	Minimal cytotoxic concentration
MIC	Minimal inhibitory concentration
NADPH	Nicotinamide adenine dinucleotide phosphate
NCBI	National Center for Biotechnology Information
NMR	Nuclear Magnetic Resonance
PD	Pharmacodynamics
PDB	Protein Data Bank
POR	Pyruvate oxidoreductase complex
PPI	Proton-pump inhibitor
PK	Pharmacokinetics
TI	Therapeutic index
Trp (W)	Tryptophan
Tyr (Y)	Tyrosine
